# Impact of Personality Traits on Knowledge Hiding: A Comparative Study on Technology-Based Online and Physical Education

**DOI:** 10.3389/fpsyg.2021.791202

**Published:** 2021-12-21

**Authors:** Jian Wu

**Affiliations:** Public Department of PE and Arts, Zhejiang University, Hangzhou, China

**Keywords:** BIG FIVE model, personality traits, psychological ownership, knowledge hiding, social status

## Abstract

Knowledge hiding has been a variable of interest that has led to major intangible losses to organizations, especially in this pandemic era when everything has shifted to online platforms and social media. Knowledge hiding has taken a new turn into the field of knowledge management. Moreover, the major players in knowledge hiding are the personality characteristics of individuals that have now found a way of expression without coming into the spotlight. This study is a necessary one in this time of online working environments where the role of personality traits and psychological ownership has been explored to understand their impact on the knowledge hiding within the organizations of China, and furthermore, to understand what role social status plays in moderating these relationships. The sampling design used is convenient random sampling with a sample size of 298 managers. This study has used the software Smart-PLS 3.3.3 for analyzing the data. The data relied on and was validated using preliminary tests of reliability and discriminant and convergent validities using the measurement model algorithm. Further, the partial least square technique was used to find the equation modeling for the variables, with the help of a structural model algorithm using 500 iterations for bootstrapping. The findings of the current study show that the personality traits of the “BIG FIVE” model positively predict knowledge hiding, except for openness to experience. At the same time, psychological ownership plays a partial mediating role.

## Introduction

The 21st century has marked drastic changes in personality grooming and behavior toward colleagues and organizations. These changes have been attributed lately to the demands of finding and retaining reputable jobs. However, such motives sometimes also develop unseen and not so socially accepted habits as well. One such challenge that organizations are facing in this technological era is knowledge hiding behavior. This study aims to understand the BIG FIVE model personality traits’ role in knowledge hiding behavior, and how psychological ownership mediates these relationships. Knowledge hiding is a process of hiding knowledge at the workplace. A situation in which a co-worker gives confidential information to others, in this case it may be misleading. That is to say; hiding is not a trick; equally, managers do not view knowledge hiding as unreliable behavior (Takala and Urpilainen, 1999).

In the 20th century, the main focus of psychologists remained the identification and measurement of personality. They keenly observed and identified personality factors that remain stable with the passage of time and keep the same influence on individuals across time and situations. Researchers always had new ideas and their new ideas compelled them to work hard and explore ever more factors of personality. In the period from 1930 to 1960, they developed a large number of tools that are very helpful for personality assessment and can measure personality traits effectively (Kondratiuk-Nierodzińska, 2016).

The BIG FIVE personality factors are five core personality traits, namely extraversion, neuroticism, conscientiousness, openness to experience, and agreeableness. In this era, personality assessment became more popular in the community of psychologists, and they tried to explore novel aspects of personality assessment. A statistical method of factor analysis was introduced to identify different domains of personality. The name of Raymond Cattell is an important one in the history of the psychology of personality, as he developed a 16 personality factor inventory (Neave et al., 2020). This concluded that personality traits are not only related to normal factors, but also related to psychopathology. Psychological ownership means that employees see the organization as a part of them, and have strong feelings and responsibilities toward the organization, thus motivating employees to take more actions that are beneficial to the organization. Ubaydullaev (2021) explained the supportive or resistive (negative) behavior by employees in the process of organizational change based on the positive and negative effects of psychological ownership. Linkov et al. (2018) analyzed that psychological ownership that is consistent with organizational goals will reduce employees’ psychological conflicts and thus facilitate the reform of enterprises. In the process of enterprise reform, the level of ownership of organizational members will increase as the degree of participation in the change increases, thereby prompting them to adopt behaviors that are conducive to change (Yuan et al., 2021).

The rationale of this research is to explore how psychological ownership mediates between personality traits and knowledge hiding. Key personality traits have been extensively used to investigate differences in person and team behavior. Research by Dawkins et al. (2015) showed that personal factors such as period of service, roles and statutes, age, gender, and personality might psychologically affect the ownership. More research is needed to examine how and to what extent the main personal difference factors may influence psychological ownership. Previous studies have investigated the predictors of psychological ownership, with little attention being paid to the effect of personality characteristics and other important personal factors (Dawkins et al., 2015). The feelings of ownership can increase by having an appropriate personality characteristic that is compliant with different motivations.

The main objective of this study is to understand the role of the BIG FIVE model personality traits in knowledge hiding behavior; and how psychological ownership mediates and social status moderates these relationships.

## Review of Literature

### Knowledge Hiding

Knowledge hiding is defined as the deliberate attempt to withhold or conceal information that another person has requested. As a result, we focus on situations where one employee made a specific request for information from another. For example, one employee may request a copy of a report from a colleague, who may respond that the information is confidential and will not disclose it. Even if no fraud is involved, the necessary details are not provided in this case.

Moreover, as with any “white lie,” the concealment of information may have good intentions or beneficial consequences. It can be used to protect another person’s feelings, keep information confidential, or protect third-party interests. As a result, it is not always wrong. In the above cases, information needs to come from individuals rather than groups or organizations. As a result, we investigate hidden information in dyads, as dyadic communication is the primary means of transmitting information within organizations (Abu Bakar and McCann, 2016).

It is also important to understand the difference between knowledge hiding and knowledge sharing (Connelly et al., 2012). When we say to hide information, it is not just a lack of sharing; rather, it is a deliberate attempt to withhold or hide information that someone else has requested. While comparisons of confidential information and sharing may lead to the belief that people share or conceal their information, we believe that these dynamics are ideologically diverse rather than contradictory. Although the two categories seem morally similar, the reasons for anonymity and lack of sharing information are very different. Information encryption can be triggered by various factors (e.g., prosocial, instrumental, laziness, etc.), while knowledge sharing is often encouraged by ignorance (Connelly and Zweig, 2015).

For example, a company employee may be approached with a request for information and opt to respond positively. However, it is presumed that he does not have the necessary knowledge to give that information at this time. Although this individual is not trying to hide this knowledge deliberately, he is having trouble participating in the process of sharing (Butt and Ahmad, 2019). Example: When an employee fails to reveal information due to a mistake, accident, or ignorance, encryption, or hiding, does not apply (Anser et al., 2021). If, on the other hand, he gets a request for information and engages in behaviors that are intended to conceal information (e.g., pretending not to have it), this is an example of concealing information.

Apart from the fact that social networking sites (SNSs) link students to the physical environment, transferring information through these sites does not go as planned. Data sharing often is not natural, because people believe that such information is important to them, leading to hidden and stored information (Bhatti et al., 2011). Previous research has shown the benefits of SNSs for knowledge sharing, but there is also the possibility that SNSs will lead students to hide their knowledge. Many areas, including organizational ethics and information management, have seen an increase in research on hidden information in recent years. However, the hidden information in online learning is not researched and developed, which requires a lot of attention. The most difficult task is to prevent students from hiding their knowledge on social media. Labafi et al. (2017) and Liu et al. (2017) investigated the controversial anonymity of information on social media sites (e.g., fear and guilt). With the emphasis on the opposition, this study was the first step in the study of hidden information in SNSs (Fang, 2017). The stimulus-organism-response (SOR) model was used to identify both objections (i.e., private concerns) and the effects of information that obscured ideas, i.e., online collaborative learning (Mehrabian and Russell, 1974; Sun et al., 2019; Zhu et al., 2020).

### History of Personality Traits

In the 20th century, the main focus of psychologists remained the identification and measurement of personality (Cornwell et al., 2020). They keenly observed and identified personality factors that remain stable with the passage of time and maintain the same influence on individuals across time and situations (Neave et al., 2020). Researchers always had new ideas and their new ideas compelled them to work hard and explore ever more facets of personality. From 1930 to 1960 they developed many tools that were very helpful for personality assessment and can measure personality traits effectively.

In this era, personality assessment became more popular in the psychological community and they tried to explore novel aspects of personality assessment. A statistical method of factor analysis was introduced to identify different domains of personality. Raymond Cattell’s name in the history of personality psychology is very important; he developed a 16-personality factor inventory. Then Hans Eysenck introduced a two factor model of personality. He argued that personality has only two dimensions extraversion and neuroticism (Fernando et al., 2019). This concluded that personality traits are not only related to normal factors, but are also related to psychopathology. After a short period, he added one more personality trait, psychoticism, in his personality model. Researchers in the 1960s tried to create a new pathway that gave a clear understanding of personality domains to every individual related to psychology. Rammstedt et al. (2010) have also done remarkable work in the field of personality.

Blanken et al. (2019) support this idea that individual behavioral differences in different situations could result from individual differences. Li and Cao (2021) also made efforts to understand the debate of nature/nurture. Cattell stressed the primacy of traits, and considered them important for personality description, starting the concept of personality testing that could measure the individual differences of individuals on the basis of traits. According to him, every individual has second-order traits. These second-order traits refer to personality traits that are commonly referred to as extroversion-introversion. The second trait is anxiety, which refers to feelings of discomfort and tension. These ideas pressed him to develop 16PF, a test of personality traits, which is used to measure 16 different personality traits.

Li and Cao (2021) researched the BIG FIVE personality test in relation to coping strategies under a stressful environment. They concluded that neuroticism is positively associated with acute stress and maladaptive coping strategies, while agreeableness and conscientiousness are negatively associated with acute stress. Relationships between brain and personality were studied. They revealed that different structures of gray and white matter, neural tracks, and their thickness had a permanent and strong association with different personality traits (Cornwell et al., 2020). Neave et al. (2020) investigated the phenomenon of emotional insecurity, inter-parental conflict, Internet addiction, and the BIG FIVE personality traits. Emotional insecurity significantly worked as a partial mediator between parental conflicts and Internet addiction. It also revealed that there were strong associations between emotionally insecure adolescents and extroverts and neuroticism.

The research conducted by Cornwell et al. (2020) concluded that poor socio-environmental resources and psychopathological factors can influence personalities apart from biological factors. A high level of neuroticism and conscientiousness were positively associated with poor socio-environmental resources. Longitudinal research investigated the association between maternal smoking status during pregnancy, education, childhood cognitive abilities of the BIG FIVE personality traits, and parental social class. Findings reveal that openness to experience, extroversion and consciousness, maternal smoking during pregnancy, and education were significant predictors of tobacco use during their lifetime.

### Extraversion and Knowledge Hiding

The most notable personality traits are extraversion and introversion. For the first time in the history of human testing both aspects have been studied. Extroverts love relationships are more comfortable with in-depth communication for the community, and show a high level of desire to associate with others. Extroverts are outgoing and confident people. Compared to the presentations, they are very enthusiastic (Arpaci et al., 2018). According to Smith et al. (2021), extroverts speak freely, are outgoing, and are comfortable interacting with the environment. Introverts are less comfortable in new environments, preferring to gain the trust of new people before interacting, and enjoy their own company. However, they may sometimes be involved in knowledge hiding (Arshad and Ismail, 2018; Issac and Baral, 2018; Pan et al., 2018). This forms the first hypothesis.

**H_1_:** Extroversion has an impact on knowledge hiding.

### Openness to Experience and Knowledge Hiding

Research was conducted to check the relationship between cyber-crime and the BIG FIVE personality model, as Silvia and Christensen (2020) revealed that there was no association between these two variables although those with high emotional stability showed less probability to feel the effects of cyber victimization. A high level of openness to experience showed the high tendency to be the victim of cyber victimization. Researchers also believe that social and personal content dynamics are more communicative (Dholariya, 2019). The characteristics of the BIG FIVE personality have been widely used in predicting problematic situations such as behavioral disorders, delinquency, and outsourcing issues (de Vries and van Prooijen, 2019) and hence knowledge hiding (Arshad and Ismail, 2018; Issac and Baral, 2018; Pan et al., 2018).

**H_2_:** Openness to experience has an impact on knowledge hiding.

### Consciousness and Knowledge Hiding

Conscience is a human trait closely related to perfectionism. The tendency to be straightforward, to plan, and to direct goals is called awareness – a force in line with community expectations. According to the analyses of psychoanalysts, people with high awareness scores have better control of stress and delay happiness. People with high levels of awareness are vigilant and cautious. They have a strong desire to do things right, they love to be guided and to follow rules and procedure, and when they are unable to do so, it makes them feel dissatisfied (Kaur and Anand, 2018). Conscience is the ability to plan and train oneself. People with high scores in this aspect are very concerned about long-term goals. A sample of 12- to 13-year-old boys who were thought to have a strong tendency toward disobedience and disruptive behavior had low knowledge (Casidy, 2012). Wisdom is a quality that has not been tested before. People who embody this feature are confident, sensible, and satisfied. They have a low level of agreeability because they do not just do things because they think they should. They make decisions based on sound logic and secular reasoning, and they stick to their own ideas and opinions though sometimes may hide something (Arshad and Ismail, 2018; Issac and Baral, 2018; Pan et al., 2018). This forms the following hypothesis:

**H_3_:** Conscientiousness has an impact on knowledge hiding.

### Neuroticism and Knowledge Hiding

Anger and anxiety are the first two facets of neuroticism; everyone experiences these two feelings sometimes in their lives. However, some people have intense feelings of fear and anger as compared to others. Neuroticism refers to experiencing negative experiences, emotions, and effects. Anxiety is the first facet of neuroticism. People who rate highly on the trait of anxiety are nervous, disorderly, and cautious (Sosnowska et al., 2019). They always feel anxious. They can derive fear from any situation and thus always feel insecure. Anger is the second facet of neuroticism. Individuals rating highly on anger and hostility express anger more frequently. They tend to be irritable and ill-tempered and may prove hard to get along with. These traits may sometimes motivate an individual to hide knowledge (Arshad and Ismail, 2018; Issac and Baral, 2018; Pan et al., 2018). This leads to the hypothesis:

**H_4_:** Neuroticism has an impact on knowledge hiding.

### Agreeableness and Knowledge Hiding

Agreeableness is a type of personality trait. People with this personality trait are kind, warm, friendly, and pliant (Templer, 2012). They have optimistic views about life, are concerned about the views of others, and they give importance to others. People who score low in agreeableness are distant, unfriendly, and uncooperative. They always put themselves first and have little concern about others. The agreeable can easily put their own interest aside for others. They are helpful friendly considerate and generous. Their beliefs about others are positive. However, they may be part of a lobby led by other individuals involved in knowledge hiding, so indirectly, they may be hiding knowledge (Arshad and Ismail, 2018; Pan et al., 2018).

**H_5_:** Agreeableness has an impact on knowledge hiding.

### Psychological Ownership Theory

This research is based on psychological ownership theory. It can be defined as the feeling of ownership or possession over an object, organization, concept, or individual that may not be reflected by the formal ownership, which may lead to the feeling of possessiveness and ownership affiliation.

For businesses to gain and maintain a competitive advantage, information is a critical source. It is significant for the company to encourage employees to share the information to get the most out of their experience. Extensive research has been directed in information management (KM) over the past two decades to determine how and why employees share information (Abubakar et al., 2019). Various organizational barriers and strategies to promote ethical sharing of information were also explored in this study. Despite all these efforts, information is still hidden from employees. When someone asks for information, concealing information refers to deliberate concealment (Lee et al., 2013). It was reported that 76% of American workers and 46% of Chinese workers participate in knowledge hiding in the workplace. Fortune 500 businesses lose an estimated $31.5 billion a year due to lack of knowledge sharing with colleagues (Jakada et al., 2021). Indeed, one of the supreme essential reasons leading to the failure of KM efforts has been identified as the secrecy of information between partners (Zhu et al., 2018). Active KM is difficult to achieve without limiting confidential information to companies (Peng and Pierce, 2015). On the other hand, previous research focused on knowledge sharing, only briefly looking at its “twin” concealing information (Peng and Pierce, 2015). Secrecy has been shown to have a negative impact on individual and organizational outcomes in a recent study. For example, reducing social support, immediately affects the performance of the individual seeking information. Workmates can retaliate if you keep your information confidential. It cripples the intelligence of the seeker and sets in motion a cycle of mistrust between partners, which leads to further secrecy. The connection between the seeker and the informant is also damaged. Because of its damaging effects on process efficiency and collaboration efficiency, secrecy of information hinders organizational performance and innovation (Zhu et al., 2018). Given the increased secrecy of information and the magnitude of its effects, it is important to study the causes of information being kept confidential in order to improve organizational involvements to reduce its recurrence. This leads to following hypotheses:

**H_6_:** Psychological ownership has an impact on knowledge hiding.

**H_7_:** Psychological ownership mediates the relationship of extroversion and knowledge hiding.

**H_8_:** Psychological ownership mediates the relationship of openness to experience and knowledge hiding.

**H_9_:** Psychological ownership mediates the relationship of conscientiousness and knowledge hiding.

**H_10_:** Psychological ownership mediates the relationship of neuroticism and knowledge hiding.

**H_11_:** Psychological ownership mediates the relationship of agreeableness and knowledge hiding.

### Social Status

The term social status is defined as the social stature of an individual in regard to prestige, honor, and their influence on achieving goals in a social circle. Importantly, it has also been defined as sacrificing one’s interests for the team’s goals and achievements (Immorlica et al., 2017). In this study, it forms a vital variable because it increases the confidence of an individual in building social relationships based on these abilities and skills. Wing Chan et al. (2011) have identified that social status plays an influential role because credibility, authenticity, and the value of the knowledge shared is judged by the social status of the individual involved. Those having high social status are considered to be authentic in their behaviors regarding knowledge. Individuals with high social status are usually considered to share their high-value knowledge for the collective goal of achievement (Rhee and Choi, 2017; Eckhardt and Bardhi, 2020). Similarly, people are less concerned with the knowledge sharing or hiding of low-status people. Hence, for this study, it is considered to be a moderating factor for knowledge hiding behavior.

**H_12_:** Social status moderates the relationship of psychological ownership and knowledge hiding.

**FIGURE 1:**
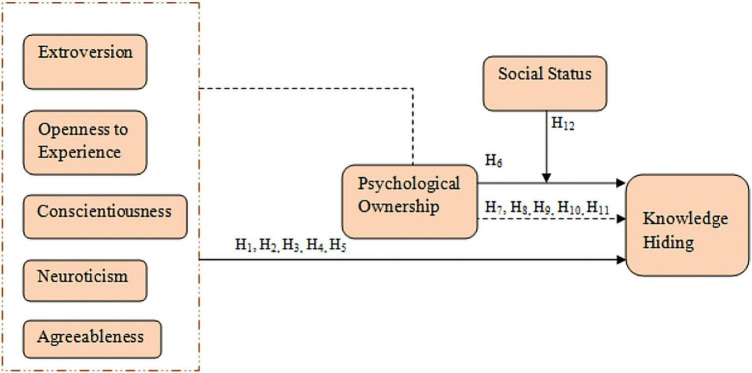
Conceptual model.

Based upon the literature review, this research was designed and the following conceptual framework (see Figure 1) was developed. The research revolves around this.

## Methodology

In this study, the impact of personality variables on knowledge hiding has been checked. Moreover, the mediating role of psychological ownership and the moderating effect of social status has also been examined. This study follows a positivist approach. Avotra et al. (2021b) have defined the positivist approach as research that examines the causes of a variable that affect another variable. In this deductive study, the hypothesis-based theory has been proposed based on the literature review done that is either validated or refused with the help of different analysis techniques. The nature of the study is cross-sectional, i.e., the data is collected once, simultaneously through structured questionnaires. The total number of items used in this study to structure the questionnaire was 37 to do the analysis. The sampling design used for this study is convenient random sampling in which the respondents are approached in accordance with the convenience of availability and accessibility. The population frame for this study was the managerial level employees of corporate organizations of China. The sample size has been obtained based on the number of items used in the questionnaire. A few questions regarding the demographics have also been included in the start of the questionnaire addressing the age, gender, and education of the employees. The technique used for data analysis was Partial least square SEM with the help of the software Smart PLS 3.3.3. Prior studies scales have been used to measure the variables of the current study.

### Instrument Development

The instrument used in this study to measure the variables has been taken from the previous study indicator scales. The scale was developed on the Likert-scale of five points where 1 = strongly disagree and 5 = strongly agree for rating the responses. The variables scales of personality traits and knowledge hiding were adapted from Peng and Pierce (2015). The questions on personality traits consisted of 19 items in total. For extroversion, openness to experience, conscientiousness, and agreeableness, the scale of each trait consisted of four items; the scale for neuroticism consisted of three items. The knowledge hiding scale consisted of eight items. Additionally, the moderation of the social status variable was adapted from Rhee and Choi (2017) and the mediating role variable of psychological ownership from Wang et al. (2019). The total items for the variable social status were six, while for mediating variable of psychological ownership, the total items were four.

## Data Analysis

Since this study has used the Smart-PLS software 3.3.3 for the data analysis, there are two main steps involved in structural equation modeling. The first stage calculates the measurement model for the validity and the reliability of the data, while the second stage measures the structural model for hypotheses testing of the relationship developed based on the theoretical framework (Avotra et al., 2021a). In this study, first, the demographic profile of the respondents has been analyzed using frequencies and percentages. The results of a demographic analysis can be seen in Table 1.

**TABLE 1:** Demographics of the respondents.

	Frequency	Percentage
**Gender**		
Male	165	55.36
Female	133	44.63
**Age**		
<20	20	6.7
21–29	89	29.86
30–39	71	23.82
40–49	104	34.89
49>	5	1.67
**Education**		
Bachelor	74	24.83
Masters	118	39.59
Doctorate	54	18.12
Others	43	14.42

*N = 298.*

**FIGURE 2:**
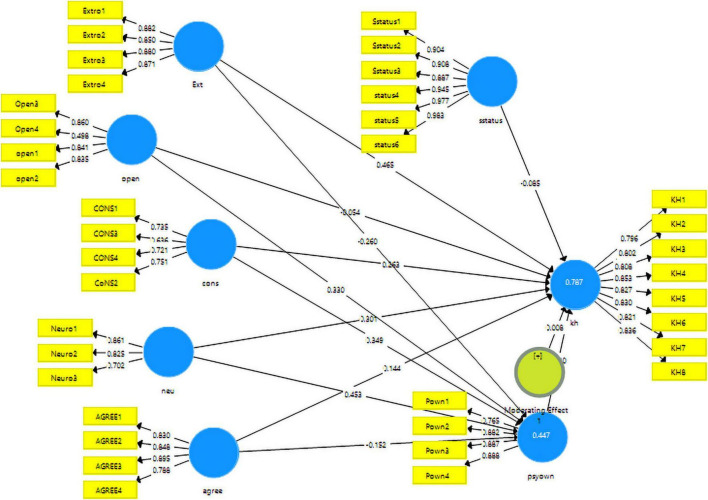
Measurement model algorithm.

This study has three steps in the data analysis. The first step has analyzed the demography of the respondents, the second stage validates the data, and in the third stage, the hypotheses were tested. This study followed the reflective-formative model. In the measurement model assessment, the data was validated using the reliabilities and the validities. The measurement model can be seen in Figure 2.

For the reliabilities, Cronbach alpha and composite reliabilities have been used. On the other hand, in order to validate the results, Fornell and Larcker criteria and heterotrait–monotrait ratio has been used. The cut-off value mentioned in the literature for reliabilities is 0.7 while for validity the average variance extracted (AVE) should be more than 0.5. Both criteria are met in this study as can be seen in Table 2.

**TABLE 2:** Constructs reliabilities and AVE.

Constructs	Code	FD	α	CR	AVE
Extroversion			0.8938	0.8958	0.7583
	Ext1	0.8750			
	Ext2	0.8596			
	Ext3	0.8810			
	Ext4	0.8673			
Openness			0.7581	0.8440	0.5766
	Open1	0.7756			
	Open2	0.7860			
	Open3	0.8156			
	Open4	0.6494			
Conscientiousness			0.6839	0.7992	0.5100
	Cons1	0.5798			
	Cons2	0.8201			
	Cons3	0.8706			
	Cons4	0.5227			
Neuroticism			0.7115	0.8386	0.6379
	Neu1	0.8816			
	Neu2	0.8481			
	Neu3	0.6460			
Agreeableness			0.8621	0.9065	0.7082
	Agr1	0.8226			
	Agr2	0.8383			
	Agr3	0.8967			
	Agr4	0.8058			
Knowledge hiding			0.9312	0.9432	0.6750
	KH1	0.8034			
	KH2	0.8139			
	KH3	0.7977			
	KH4	0.8533			
	KH5	0.8260			
	KH6	0.8273			
	KH7	0.8161			
	KH8	0.8335			
Psychological ownership			0.8812	0.9195	0.7421
	PO1	0.7281			
	PO2	0.8997			
	PO3	0.9004			
	PO4	0.9046			
Social status			0.9709	0.9765	0.8740
	SS1	0.9040			
	SS2	0.9083			
	SS3	0.8871			
	SS4	0.9454			
	SS5	0.9769			
	SS6	0.9832			

*N = 298. AVE, average variance extracted; CR, composite reliability; FD, factor loading.*

Moreover, to further validate the data collected, the HTMT ratio and Fornell and Larcker criterion were also calculated in the study. The values in the Fornell and Larcker should be highest at the top of each column. This can be validated and seen in Table 3 which gives the figures of the Fornell and Larcker test for the validity of the data.

**TABLE 3:** Fornell and Larcker criterion.

	Agree	Cons	Ext	KH	Neuro	Open	Sstatus	psyown
Agree	0.842							
Cons	0.447	0.714						
Ext	0.519	0.553	0.871					
KH	0.596	0.546	0.801	0.822				
Neuro	0.532	0.569	0.604	0.722	0.799			
Open	0.647	0.484	0.594	0.490	0.401	0.759		
Sstatus	0.202	0.705	0.265	0.208	0.288	0.403	0.935	
psyown	0.364	0.629	0.366	0.351	0.577	0.452	0.665	0.862

*N = 298.*

*Cons, conscientiousness; Ext, extroversion; KH, knowledge hiding; Neuro, neuroticism; Open, openness to experience; Sstatus, social status; psyown, psychological ownership.*

Additionally, the HTMT ratio mentioned in the Table 4 has also validated the data with values around 0.9. The values above 0.9 may indicate the problem of multicollinearity if the reliabilities have not been met. However, this study meets the reliability and validity criteria using Cronbach alpha and composite reliability for reliabilities and Fornell and Larcker criterion and HTMT ratios for validities, hence no issues of multicollinearity may arise as Tables 2–4 illustrate the uniqueness of each variable in measuring their own constructs.

**TABLE 4:** HTMT ratio.

	Agree	Cons	Ext	KH	Neuro	Open	Sstatus	psyown
Agree								
Cons	0.677							
Ext	0.585	0.836						
KH	0.659	0.844	0.880					
Neuro	0.711	0.968	0.778	0.908				
Open	0.828	0.700	0.751	0.603	0.567			
Sstatus	0.221	0.766	0.283	0.217	0.346	0.432		
psyown	0.406	0.758	0.394	0.367	0.703	0.516	0.733	

**FIGURE 3:**
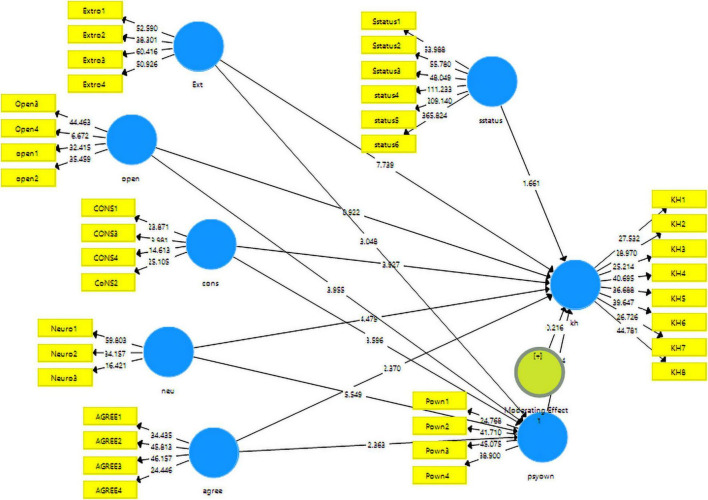
Structural model algorithm.

In the second phase of structural equation modeling, the hypotheses developed in the theoretical framework were checked to establish whether they supported the data or not. This phase of SEM includes the direct and indirect relationships. The direct relationships check the direct impact of a variable on another variable. However, in indirect relationships, the mediation of a certain variable is checked against the relationships of two other independent and dependent variables. In this study, the estimation results of beta, *t*-statistic, *p*-values, and adjusted *R*^2^ have been used to accept or reject the hypotheses of the study. The algorithm for the structural model has been added in the study with Figure 3.

These results obtained from the structural model assessment can be seen in Table 5. There were 12 hypotheses in total, of which six hypotheses were accepted while others were rejected. Among the direct relationships hypothesized in this study, only openness to experience did not find any impact on the knowledge hiding *t*-statistic = 0.922, *p*-value = 0.357, thus rejecting H_2_. However, the rest of the direct relationships were found as significantly having a positive contribution to knowledge hiding. Extroversion was found to be positively and significantly impacting the knowledge hiding with *t*−*statistic* = 7.739:*p*−*value* = 0.000, accepting H_1_. In regard to H_3_, conscientiousness was also found to have a significant impact on knowledge hiding *t*−*statistic* = 3.927:*p*−*value* = 0.000. Furthermore, neuroticism *t*−*statistic* = 4479:*p*−*value* = 0.000 and agreeableness *t*−*statistic* = 2.370:*p*−*value* = 0.018, thus supporting the H_4_ and H_5_ developed from the literature. Psychological ownership also found a significant role in knowledge hiding, *t*−*statistic* = 2.244:*p*−*value* = 0.025. Among the indirect effects of personality traits with knowledge hiding taking into account the mediation of psychological ownership, none of the relationships could find a significant mediating contribution of psychological ownership except neuroticism *t*−*statistic* = 2.035:*p*−*value* = 0.042, thus accepting H_10_. The rejected hypotheses of mediation H_7_, H_8_, H_9_, and H_11_ were rejected with a small margin of insignificance. Moreover, regarding the moderation effect of social status in the relationship of psychological ownership and knowledge hiding, this could not be statistically approved hence, rejecting H_12_. These results can be seen in Table 5.

**TABLE 5:** Results for structural model.

Paths	H	O	*M*	SD	*t*-Stats	*p*-Value	Results
Ext → KH	H_1_	0.465	0.460	0.060	7.739	0.000	Accepted
Open → KH	H_2_	−0.054	−0.049	0.058	0.922	0.357	Rejected
Cons → KH	H_3_	0.263	0.267	0.067	3.927	0.000	Accepted
Neu → KH	H_4_	0.301	0.301	0.067	4.479	0.000	Accepted
Agree → KH	H_5_	0.144	0.141	0.061	2.370	0.018	Accepted
PO → KH	H_6_	−0.110	−0.107	0.049	2.244	0.025	Accepted
Ext → PO → KH	H_7_	0.029	0.028	0.016	1.809	0.071	Rejected
Open → PO → KH	H_8_	−0.036	−0.036	0.020	1.852	0.065	Rejected
Cons → PO → KH	H_9_	−0.038	−0.037	0.021	1.833	0.067	Rejected
Neu → PO → KH	H_10_	−0.050	−0.050	0.025	2.035	0.042	Accepted
Agr → PO → KH	H_11_	0.017	0.017	0.012	1.393	0.164	Rejected
SsMod → KH	H_12_	0.008	0.006	0.036	0.216	0.829	Rejected

*N = 298.*

*H, hypotheses; O, original sample; M, sample mean; SD, standard deviation; Cons, conscientiousness; Ext, extroversion; KH, knowledge hiding; Neuro, neuroticism; Open, openness to experience; SsMod, social status as moderator; PO, psychological ownership.*

## Discussion

This research aims to evaluate the proposed connection between knowledge hiding and personality traits from the perspective of online learning. In today’s world, information management systems are an integral part of every organization. Information management systems are seen as a channel through which organizations can extract, exchange and use information (Zhu et al., 2018).

It was hypothesized that extroversion has an impact on knowledge hiding. Findings from this study revealed that extraversion strongly predicts knowledge hiding. People who are extroverts can successfully hide their knowledge in online academia (Templer, 2012). The majority of research examining the prevalence of BIG FIVE characteristics in entrepreneur and management populations was conducted between 1960 and 2000 when most of the data was gathered. Because both entrepreneurs and managers can potentially supervise employees and manage various responsibilities, managers are commonly used as a real comparison.

It was also hypothesized that openness to experience has an impact on knowledge hiding. The findings of the study reject this hypothesis. People are open to the experience as they love to share their knowledge with others. It was also hypothesized that consciousness, neuroticism, and agreeableness have an impact on knowledge hiding. The findings of the study revealed that all these personality traits positively predict knowledge hiding. Previous studies also support the findings of this research. Abdellaoui et al. (2019) found that firms are much more open to new experiences, especially considerate, average for openness to experience, less agreeable, and much less emotionally unstable (or in the BIG FIVE lingo, O+, C+, E, A−, N−) in a meta-analysis of 23 research studies from 1970 to 2002 in foreign nations and noted in English-language journal articles.

Numerous investigations have shown that this trend is not always followed. There is evidence to support this, for instance, Kroencke et al. (2020) found that in a survey of 218 business owners and managers in a major Canadian city, employees were less diligent and pleasant than business owners and less extraverted (O+) than business managers. To explain these distinctions between entrepreneurship and the average employee, researchers often use the “attraction–selection–attrition model.” As a result of this model, employees are drawn to jobs whose requirements and possibilities match their innate talent and motivations. At the same time, companies and investors select candidates who meet their criteria. Employees then stay in their area of employment when they discover their professional scenario is more enjoyable than alternative employment options. Entrepreneurs have a greater willingness to learn from mistakes than managers do. Entrepreneurs, according to researchers, are drawn to continually changing environments and innovative problems because of the novelty (Ali et al., 2021). Entrepreneurs’ willingness to take on new problems and work in unique contexts may help individuals who offer innovative solutions, business methods, and goods. As a result, instead of seeking for novel ideas, managers are typically chosen by their bosses for their capacity to carry out and provide consistent, high-quality work with low variation for a particular set of guidelines. Because of this, studies believe that entrepreneurship attracts people who are more willing to try new things. According to Tong (2010) higher conscientiousness is by far the most substantial difference among entrepreneurship. Agreeableness is a combination of a drive for success and the ability to rely on one’s own abilities (Kaur and Anand, 2018). When it comes to dependability, successful entrepreneurs are about the same, but employees come out on top when it comes to accomplishment (Beaty et al., 2016). For example, (Watson et al., 2019) found that entrepreneurs have higher levels of achievement motivation than other workers. Spark and O’Connor (2021) also found that entrepreneurs have higher levels of achievement motivation than managers.

A common theory holds that high achievers prefer working in circumstances where their successes are directly linked with their efforts rather than in a bigger institutional context where commercial accomplishment is less directly linked to one’s own efforts. There is little agreement on whether entrepreneurs have a greater extraversion score than managers. Research suggests extraversion is more crucial for entrepreneurs since they sell their ideas to potential investors, partners, workers, and customers as salesmen. There is, however, no conclusive difference found by Mann et al. (2017) in the research. According to Fang (2017), entrepreneurs are less extroverted than managers, which suggests that many business owners run their small enterprises from their residences to avoid the massive bureaucracy that requires one to be obsessively social. Self-employed people and growth-oriented founders exhibit extremely different qualities depending on how you define an “entrepreneur.” When it comes to cooperativeness and psychoticism (A−, N−), employees tend to score lower than managers on average. According to some theories, entrepreneurs don’t have to worry about impressing others because they will ultimately get to be the executives of their very own companies. In contrast, managers must at the very least, properly respect their superiors.

It was also hypothesized that psychological ownership has an impact on knowledge hiding (Ma et al., 2020). The findings of the study reject this hypothesis. It was also hypothesized that psychological ownership mediates the relationship between extroversion and knowledge hiding. The findings of the study reject this hypothesis. It was also hypothesized that psychological ownership mediates the relationship of openness to experience and knowledge hiding. The findings of the study reject this hypothesis (Butt and Ahmad, 2019). It was also hypothesized that psychological ownership mediates the relationship between conscientiousness and knowledge hiding. The findings of the study reject this hypothesis. It was also hypothesized that psychological ownership mediates the relationship between neuroticism and knowledge hiding. The findings of the study accept this hypothesis. It was also hypothesized that psychological ownership mediates the relationship of agreeableness and knowledge hiding. The findings of the study reject this hypothesis. It was further hypothesized that social status moderates the relationship between psychological ownership and knowledge hiding. The findings of the study reject this hypothesis. Previous studies supported the findings of this research. Differing characteristics were found among self-employed people in recent research, while similar characteristics are being measured among various aspects of entrepreneurship or varying degrees of purpose. All these differences are interesting and policy significant on a macro-level as representations of entrepreneurs against the average individual (Connelly and Zweig, 2015). There were six face-to-face interviews with business owners at companies and 501 evaluations filled out by students at Slovenian academic institutions, resulting in the classification of individuals into four groups: entrepreneurs who are already in business (20% of responses); entrepreneurs who plan to start a business in the next 3 years (9.9%); entrepreneurs who are thinking about starting a company in the market (42.4%); and non-entrepreneurs (the remaining 27.7%). Researchers found variations that mirrored the conceptual findings for openness: businesspeople who are already in business are the most receptive to learning from their mistakes, whereas aspiring entrepreneurs are slightly more closed off. When it comes to agreeableness, small business owners are the least agreeable of all. However, meta-analyses have found no correlation between conscientiousness and agreeableness or psychoticism.

According to Anser et al. (2021), business owners are much more willing to try new things than the general population. However, other characteristics are more difficult to pin down, and the remaining ambiguity can be explained thus. Quantitative measurements, which often only catch a few hundred people, account for much of the diversity across research (Zhai et al., 2021). Meta-analyses are limited because of the influence of environmental factors on the entrepreneurial traits of each community, making cross-population, industry, and cultural generalizations unfeasible. We may be able to distinguish between the clutter of tiny amounts and the real variations in entrepreneurial personality traits across situations if more studies are undertaken, which would be a big accomplishment. An additional criticism of the BIG FIVE framework is that these macro personality traits are overly general, making it difficult to forecast entrepreneurial behavior based on specific situational circumstances. Furthermore, understanding a person’s BIG FIVE character may not assist in knowing the exact mechanism by which their identity influences their entrepreneurship actions and attitudes (Chen et al., 2020).

Owing to this lack of satisfaction and the inability to paint a complete picture of the entrepreneurial human, BIG FIVE researchers turned in developing a 13-dimensional personality framework that includes other traits including consciousness, organizational innovation, locus of control, and the desire to succeed. These are the things we will go through next. Furthermore, it’s not uncommon for this to be done on purpose (Fang, 2017). Information sharing is widely regarded as essential for knowledge refinement, generation, and individual recognition. Ma et al. (2020) found that it is frequently overlooked. According to a study conducted on Chinese skilled professionals, 59% of them engage in information concealment behavior (Neave et al., 2020). The essential principles of organizational learning including “knowledge sharing,” “expertise withholding,” and “expertise stockpiling” must not be overlooked in order to grasp the knowledge concealing architecture. Different organizations have diverse attitudes on the sharing of knowledge. Some people believed that sharing should be discouraged because it could leak company secrets, while others see tremendous value in disseminating information within a company. This term describes the process of exchanging information (knowledge) between people and groups, organizational units, as well as organizations themselves (Beaty et al., 2016). In the absence of information exchange, organizations would be forced to perpetually reinvent themselves to take advantage of previous experiences and expertise they could have used (Connelly and Zweig, 2015). As a part of the information transfer phenomena, they argue that “knowledge concealing,” “information hoarding,” and “information sharing” are all interconnected. The authors characterized a person’s deliberate attempt to conceal or hide information demanded by another person as a knowledge concealment structure (Avotra et al., 2021a). Information holding, a similar notion, differs from information concealing in that an individual might be unable to communicate the knowledge due to error or ignorance in some cases. Whilst some people are more inclined to be chatty, others may have a willingness to give knowledge. There are those who believe that knowledge is power, which they most likely learned in workplaces. These individuals may hoard understanding and be unwilling to share it, which leads to inefficiency and provider separation (Jonason et al., 2017).

There are numerous reasons why knowledge transmitters keep their expertise close to their chests. One issue is the risk of losing market value after putting in years of study and training. As a result, employees have a strong sense of ownership over the gathered knowledge. The second argument is that information sharing is expensive and adds additional responsibilities or burdens to those disseminating the knowledge on top of their usual responsibilities. A third factor is a fear of harboring “knowledge parasites” that have not put in as much work as the potential knowledge sharer has. Fourth, they avoid having their knowledge evaluated by others. Finally, subordinates deliberately hoard information in the belief that superiors dislike knowledgeable subordinates who know more or think they know more than their superiors. On the other hand, Superiors may purposefully withhold information from colleagues to maintain the competitive advantage of authority (Watson et al., 2019). Knowledge concealment is motivated by a variety of factors. Some of them cover personal causes like routine and laziness, whereas cultural aspects are also mentioned (Smith et al., 2021).

## Conclusion

Knowledge hiding has been one of the most studied variables in knowledge management. Although knowledge hiding is considered a negative human behavior it is not always harmful. Sometimes individuals hide their knowledge in order to gain some professional advantages over their colleagues, or sometimes for personal interests. However, the role of the nature of the person has been overlooked in literature in understanding the role of knowledge hiding. Therefore, this study has proposed the role of personality traits in the knowledge hiding behaviors of individuals. Among the five traits of the BIG FIVE model of personality, all personality traits except for openness to experience, have been found to have a significant contribution in knowledge hiding behavior. However, the psychological contract is found to mediate only the relationship of neuroticism and knowledge hiding. The significant results show that the attainment of individuals’ inner motives makes them hide their knowledge and exploit it for their best use. Another objective of knowledge hiding is when individuals spend a long time gaining knowledge, they feel reluctant in sharing with others because they tend to feel that they own it as they have spent their valuable years acquiring it. There are many other factors that strongly contribute to knowledge hiding, such as psychological pressure and the workplace environment. In future research, these factors should also be explored from the perspective of knowledge hiding.

## Data Availability Statement

The original contributions presented in the study are included in the article/supplementary material, further inquiries can be directed to the corresponding author.

## Ethics Statement

The studies involving human participants were reviewed and approved by Zhejiang University, China. The patients/participants provided their written informed consent to participate in this study. The study was conducted in accordance with the Declaration of Helsinki.

## Author Contributions

JW conceived and designed the concept, collected the data, and wrote the manuscript. The author read and agreed to the published version of the manuscript.

## Conflict of Interest

The author declares that the research was conducted in the absence of any commercial or financial relationships that could be construed as a potential conflict of interest.

## Publisher’s Note

All claims expressed in this article are solely those of the authors and do not necessarily represent those of their affiliated organizations, or those of the publisher, the editors and the reviewers. Any product that may be evaluated in this article, or claim that may be made by its manufacturer, is not guaranteed or endorsed by the publisher.
